# Trivalent Adenovirus Type 5 HIV Recombinant Vaccine Primes for Modest Cytotoxic Capacity That Is Greatest in Humans with Protective HLA Class I Alleles

**DOI:** 10.1371/journal.ppat.1002002

**Published:** 2011-02-24

**Authors:** Stephen A. Migueles, Julia E. Rood, Amy M. Berkley, Tiffany Guo, Daniel Mendoza, Andy Patamawenu, Claire W. Hallahan, Nancy A. Cogliano, Nicole Frahm, Ann Duerr, M. Juliana McElrath, Mark Connors

**Affiliations:** 1 Laboratory of Immunoregulation, National Institute of Allergy and Infectious Diseases, National Institutes of Health, Bethesda, Maryland, United States of America; 2 Vaccine and Infectious Disease Division and the HIV Vaccine Trials Network, Fred Hutchinson Cancer Research Center, Seattle, Washington, United States of America; 3 Biostatistics Research Branch, National Institute of Allergy and Infectious Diseases, National Institutes of Health, Bethesda, Maryland, United States of America; Harvard University, United States of America

## Abstract

If future HIV vaccine design strategies are to succeed, improved understanding of the mechanisms underlying protection from infection or immune control over HIV replication remains essential. Increased cytotoxic capacity of HIV-specific CD8^+^ T-cells associated with efficient elimination of HIV-infected CD4^+^ T-cell targets has been shown to distinguish long-term nonprogressors (LTNP), patients with durable control over HIV replication, from those experiencing progressive disease. Here, measurements of granzyme B target cell activity and HIV-1-infected CD4^+^ T-cell elimination were applied for the first time to identify antiviral activities in recipients of a replication incompetent adenovirus serotype 5 (Ad5) HIV-1 recombinant vaccine and were compared with HIV-negative individuals and chronically infected patients, including a group of LTNP. We observed readily detectable HIV-specific CD8^+^ T-cell recall cytotoxic responses in vaccinees at a median of 331 days following the last immunization. The magnitude of these responses was not related to the number of vaccinations, nor did it correlate with the percentages of cytokine-secreting T-cells determined by ICS assays. Although the recall cytotoxic capacity of the CD8^+^ T-cells of the vaccinee group was significantly less than that of LTNP and overlapped with that of progressors, we observed significantly higher cytotoxic responses in vaccine recipients carrying the HLA class I alleles B*27, B*57 or B*58, which have been associated with immune control over HIV replication in chronic infection. These findings suggest protective HLA class I alleles might lead to better outcomes in both chronic infection and following immunization due to more efficient priming of HIV-specific CD8^+^ T-cell cytotoxic responses.

## Author Summary

Unique HIV-infected individuals have remained healthy with stable CD4 counts and HIV RNA levels below the detection threshold in sensitive assays without antiretroviral therapy for 20 years. These nonprogressors have been intensively studied in order to identify mechanisms that could inform the design of an efficacious HIV/AIDS vaccine. In addition to strong associations with certain host genes like HLA B*57, nonprogressors are distinguished from progressors by the superior ability of their HIV-specific CD8^+^ T-cells to proliferate and to efficiently kill HIV-infected CD4^+^ T-cell targets via perforin and granzyme B, the major proteins contained within killing granules. Here, for the first time, we apply sensitive measurements of CD8^+^ T-cell proliferation and perforin expression, granzyme B target cell activity and infected CD4^+^ T-cell elimination to samples derived from recipients of the Merck adenovirus serotype 5-HIV vaccine. We demonstrate readily detectable CD8^+^ T-cell-mediated killing in these vaccinees. Although the killing responses were less than those of nonprogressors, vaccinees expressing the protective HLA alleles B*27, B*57 or B*58 exhibited greater killing than those not possessing these alleles. These findings suggest protective HLA alleles lead to better outcomes in both chronic infection and following immunization through early interactions that induce superior antiviral CD8^+^ T-cell killing responses.

## Introduction

Understanding the fundamental basis of immunologic control of HIV remains an enormous challenge in the development of efficacious HIV vaccines and immunotherapies. Some important clues have emerged from studies of rare patients with natural immune control over HIV referred to as long-term nonprogressors (LTNP), HIV controllers, elite suppressors or elite controllers who contain HIV replication for many years to less than 50 copies/mL plasma without antiretroviral therapy (ART) (reviewed in [1]). Several lines of evidence suggest that HIV-specific CD8^+^ T-cell responses are responsible for mediating immune control in these individuals. Among these are strong, consistent associations between nonprogressive infection and particular HLA class I alleles like B*57 [2]–[8]. In B*57^+^ LTNP, this genetic association is paralleled by functional data demonstrating an overwhelming immunodominance of HLA B57-restricted, HIV-specific CD8^+^ T-cells [2], [9], [10]. Similar observations between protective MHC alleles, like Mamu B*08 and B*17, and prolonged restriction of SIV replication have been made in the rhesus macaque model of SIV infection [11]–[13]. Greater insight into the mechanisms underlying these associations, which are among the strongest observed in human diseases as determined by a number of approaches, will certainly enhance our understanding of the parameters necessary for the induction and/or maintenance of immune-mediated control of HIV infection.

Recently, several important advances have been made in understanding the mechanism of immunologic control of HIV in humans. It has been known for some time that patients with immunologic control are not distinguished by greater frequencies or breadth of HIV-specific CD8^+^ T-cells or by the particular specificities that are targeted [2], [14]–[16]. These observations have suggested that the CD8^+^ T-cells of LTNP are not differentiated from those of progressors on the basis of quantitative considerations. HIV-specific CD8^+^ T-cells of LTNP have been observed to mediate a greater number of functions based upon cytokine and chemokine secretion compared to progressors, although there is considerable overlap between these patient groups [17]–[19]. Most notably, the CD8^+^ T-cells of LTNP have been distinguished from those of progressors based upon their ability to suppress HIV replication *in vitro* or in humanized mice [5], [20]. The mechanism underlying this suppressive capacity is the greater ability of LTNP CD8^+^ T-cells to expand and produce the pore-forming molecule perforin, which is necessary for granule-exocytosis mediated cytotoxicity [8], [19], [21]. HIV-specific CD8^+^ T-cells of LTNP possess extraordinary cytotoxic capacity against primary HIV-infected target cells on a per-cell basis measured by granzyme B target cell activity and infected CD4^+^ T-cell elimination [8], [19]. This is a function that clearly differentiates these individuals from untreated or treated patients without immune-mediated control of HIV. High levels of cytotoxic capacity are not recovered when the level of antigen is reduced through antiretroviral therapy [19]. Although this function clearly distinguishes those with immunologic control in the setting of chronic infection, it may not necessarily be the operative mechanism in control induced by an HIV vaccine. Thus far, direct measurements of recall cytotoxic capacity assessed by granzyme B target cell activity and infected CD4^+^ T-cell elimination have not been applied to recipients of HIV vaccines.

Although a number of vaccine candidates under development aim to induce cellular immune responses directed against HIV, those among the most immunogenic based upon pre-clinical and phase I clinical studies have been the replication incompetent adenovirus serotype 5 (Ad5) HIV-1 recombinant vaccines [22]–[25]. An efficacy trial employing such a vaccine was the Step study, a phase IIB test-of-concept trial involving 3,000 HIV-negative individuals at high risk of HIV infection [26], [27]. After one year of follow up, HIV RNA levels were comparable among those who became infected regardless of immunization [26]. Initial analyses, using a validated interferon-γ ELISPOT assay and an intracellular cytokine staining (ICS) assay, revealed no differences in vaccine-induced HIV-specific immunity, including response rate, magnitude and cytokine profile, between male cases and non-cases [27]. These findings suggested that if vaccine-induced immunologic control is to succeed, future candidate vaccines may need to elicit responses of higher magnitude or different breadth or function.

In addition to these findings, some data have suggested an impact of host HLA in response to vaccination. CD8^+^ T-cells restricted by protective alleles were observed to dominate the response to vaccination with an ALVAC-HIV recombinant canarypox [28]. In addition, in one early analysis of the Step study, more individuals carrying HLA class I alleles that have been associated with immune control in the chronic phase of infection like B*57 and B*27, who subsequently became infected with HIV, were controlling HIV replication to low levels (Nicole Frahm, personal communication) although, in a more recent analysis, this did not achieve statistical significance. These data are preliminary and the numbers are small. It is important to note that vaccinee cases were not distinguished by quantitative measurements of their HIV-specific CD8^+^ T-cell response by ELISPOT (Nicole Frahm, personal communication).

In the present study, we examined the HIV-specific CD8^+^ T-cell cytotoxic capacity of HIV-1-uninfected recipients of the Merck Ad5/HIV trivalent vaccine. We observed CD8^+^ T-cells in some vaccinees exhibiting proliferative capacity and the ability to upregulate perforin expression that overlapped with those of LTNP. However, the ability of these cells to kill primary autologous HIV-infected targets was relatively low and comparable to that of progressors. This low cytotoxic capacity was not related to precursor or effector cell frequencies. Although this cytotoxic capacity was modest in most vaccinees, the highest responses were observed in those with the protective HLA class I alleles B*57, B*58 and B*27. Taken together these data suggest that the Merck Ad5/HIV trivalent vaccine induced relatively modest cytotoxic capacity against HIV-1-infected cells in vaccinees. In addition, they suggest that protective HLA class I alleles have an effect on the cytotoxic capacity induced by vaccination that is mediated during T-cell priming.

## Materials and Methods

### Ethics statement

For this study, approval to transfer samples from immunized Human Immunodeficiency Virus (HIV)-seronegative participants of HIV Vaccine Trials Network (HVTN) protocols 071 or 502 was granted by the Fred Hutchinson Cancer Research Center Institutional Review Board (IRB# 00000022). HIV-infected subjects and non-immunized HIV-seronegative controls were recruited from the Clinical Research Center, National Institutes of Health (Bethesda, MD) and signed National Institute of Allergy and Infectious Diseases Investigational Review Board (NIAID IRB#5)-approved protocol informed consent documents. The study was conducted according to the principles expressed in the Declaration of Helsinki.

### Study subjects

As part of HVTN protocols 071 or 502, 31 HIV-negative individuals received 2 (n = 19) or 3 (n = 12) immunizations, respectively, of the Merck recombinant, replication-incompetent adenovirus serotype 5 (Ad5) HIV-1 trivalent vaccine, which was a mixture of three E1-deleted recombinant Ad5 viruses, each containing one of three HIV-1 inserts (*gag, pol* and *nef*), as described previously [27]. The vaccine was administered intramuscularly as a 1-mL injection of 1.5×10^10^ adenovirus genomes. The vaccine recipients underwent large volume venipuncture or leukapheresis a median of 331 (range, 8–1,315) days after the last vaccination at the site of enrollment. The first 19 samples, which were randomly selected, contained cells from only 5 individuals with the protective HLA class I alleles B*27, B*57 or B*58. Therefore, 12 additional samples, including cells from 6 patients with protective HLA class I alleles, were provided. In total, 11 vaccinees carried one of these protective HLA class I B alleles versus 20 vaccinees who did not (Table S1). Investigators performing the studies were blinded to the HLA haplotype of the vaccinees. HLA class I typing was performed by a high-throughput sequencing based PCR method as described previously [29].

HIV infection was documented by HIV-1/2 immunoassay in LTNP, viremic progressors and antiretroviral therapy (ART)-treated progressors who were defined as described previously (Table S2) [8], [19]. ART-treated progressors (Rx<50) received continuous ART and had HIV RNA levels suppressed to <50 copies/ml for a median of 8 (range 5–9) years. Median CD4^+^ T-cell counts for LTNP, viremic progressors and Rx<50 were 955 (range 664–1,362), 449 (range 238–739) and 692 (range 408–720) cells/ml, respectively. Since HIV-specific CD8^+^ T-cell functionality with respect to proliferative and cytotoxic capacities has been shown to be similar between viremic progressors and Rx<50 [8], [19], these subgroups were combined into a single progressor group in the statistical analyses. Peripheral blood mononuclear cells (PBMC) were obtained as described previously [21]. HLA class I/II typing was performed by sequence-specific hybridization as described previously [2].

### HIV_SF162_-infected autologous CD4^+^ T-cell targets

CD4^+^ T-cells were positively selected from cryopreserved PBMC derived from vaccinees and chronically infected patients by magnetic automated cell sorting (AutoMACS, Miltenyi Biotec, Germany) and polyclonally stimulated with medium containing anti-CD3 (Orthoclone OKT3, 1 µg/ml; Ortho Biotech, Bridgewater, NJ), anti-CD28 (1 µg/ml, BD Biosciences) and human IL-2 (40 IU/ml, Roche Diagnostics, Indianapolis, IN) prior to infection as previously described [21]. CD4^+^ lymphoblasts were infected over 24–36 hours as recently described [8], [19], [30]. The percent infection of CD4^+^ T-cell targets was confirmed in all cases by intracellular HIV-1 Gag p24 expression using flow cytometry and was similar among LTNP (median 59.9%), progressors (56.9%), HIV-negative controls (60.5%) and vaccine recipients (69.4%).

### Granzyme B cytotoxicity assay and infected CD4^+^ T-cell elimination (ICE)

Day 6 effector cells (PBMC incubated with infected targets for 6 days) were labeled with immuno-magnetic beads (CD8^+^ T-cell Isolation Kit II, Miltenyi Biotec) prior to negative selection of CD8^+^ T-cells by magnetic automated cell sorting as described previously [8]. Cytotoxic responses were measured against LIVE/DEAD Fixable Violet Stain (Molecular Probes, Invitrogen Detection Technologies, Eugene, OR, USA)-labeled HIV_SF162_- infected and uninfected autologous CD4^+^ T-cell targets in assays examining GrB target cell activity and infected CD4^+^ T-cell elimination (ICE) as recently reported [8], [19]. To assess per-cell cytotoxic capacity, ICE responses were measured at a standard E:T ratio of 25∶1 (total day 6 CD8^+^ T-cells to total CD4^+^ T-cell targets) for all individuals, and additionally at an E:T ratio of 50∶1 in 20 vaccinees from whom greater numbers of PBMC were available. These responses were then plotted against the true E:T ratios, which were determined by measurements of IFN-γ-secreting CD8^+^ T-cells and p24-expressing target cells (sum of CD4^-^p24^+^ and CD4^+^p24^+^ cells in plots containing only infected targets), respectively, as previously described [8], [19].

### CFSE proliferation assays

PBMC were labeled with 5,6-carboxyfluorescein diacetate, succinimidyl ester (CFSE; Molecular Probes, Eugene, OR) and incubated with medium, anti-CD3 (Orthoclone OKT3, 1 µg/ml) and anti-CD28 (1 µg/ml) antibodies or uninfected or HIV_SF162_-infected autologous CD4^+^ T-cell targets in 96-well, deep-well culture plates (PGC Scientifics, Frederick, MD) at a density of 10^6^ PBMC/well/ml for 6 days as previously described [21].

### CD8^+^ T-cell stimulation assays for intracellular protein detection

In experiments using CD4^+^ T-cell targets to measure the total frequency of virus-specific CD8^+^ T-cells in cytotoxicity experiments, negatively selected CD8^+^ T-cells (incubated with HIV_SF162_-infected targets for 6 days) were co-incubated with uninfected or HIV_SF162_-infected autologous CD4^+^ T-cell targets for 6 hours prior to fixation, permeabilization and intracellular IFN-γ staining as described previously [8], [19].

In flow cytometric intracellular cytokine staining (ICS) assays employed to enumerate HIV-specific T-cells in vaccine recipients, thawed PBMC were cultured overnight and then stimulated for 6 hours with HIV-1 Nef, Gag or Pol peptide pools that span the sequence encoded by the HIV-1 gene inserts in the vaccine in order to detect IFN-γ and IL-2 production by CD8^+^ and CD4^+^ T-cells, as described previously [27]. Pools of potential T-cell epitope (PTE) 15-mer peptides were used in the 071 study and pools of 15-mer peptides overlapping in sequence by 11 amino acids were used in the 502 study. The criteria for positive and negative responses were defined previously [31].

### Flow cytometry

Multiparameter flow cytometry was performed according to standard protocols. Surface and/or intracellular staining was done using the following antibodies from BD Biosciences, unless otherwise noted: fluorescein isothiocyanate (FITC)-conjugated anti-CD3; peridinine chlorophyll protein (PerCP)-conjugated anti-CD3 and anti-CD4; allophycocyanin (APC)-conjugated anti-CD4, anti-CD8 and anti-IFN-γ; APC H7-conjugated anti-CD3; phycoerythrin (PE)-conjugated anti-CD8; Pacific Blue-conjugated anti-perforin and RDI-conjugated anti-p24 antibodies (Kc57, Beckman Coulter, Inc., Fullerton, CA). Unless otherwise specified, all staining was performed at 4°C for 30 minutes. In cytotoxicity experiments, gates were drawn on labeled CD4^+^ T-cell targets and 5,000-8,000 events were collected. Samples were analyzed on a FACSAria multi-laser cytometer (Becton-Dickinson) with FACSDiva software. Color compensations were performed using single-stained samples for each of the fluorochromes used. Data were analyzed using FlowJo software (TreeStar, San Carlos, CA).

### Statistical analysis

The Wilcoxon signed rank test was used to compare paired data. Independent groups were compared by the Wilcoxon two-sample test. Correlation was determined by the Spearman rank method. The Bonferroni method was used to adjust p values for multiple testing. Regression analysis, analysis of covariance and repeated measures were used to quantify the differences in ICE among LTNP, viremic progressors and vaccine recipients over the common ranges of logged E:T ratios, at the median logged E:T ratio of the combined independent groups and at the logged E:T ratio of 5∶1.

## Results

### HIV-specific CD8^+^ T-cell cytotoxic responses induced by an Ad5/HIV vaccine are similar to those of progressors

**Figure 1:**
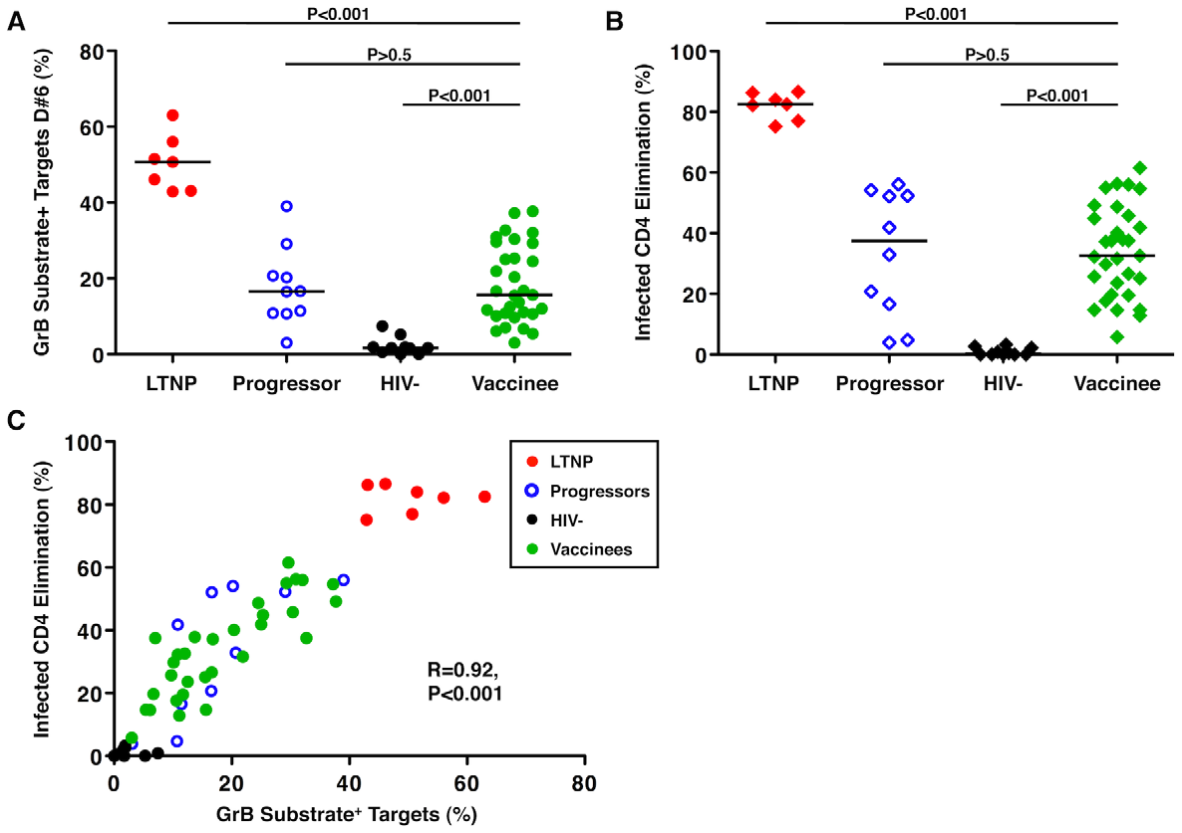
HIV-specific CD8^+^ T-cell cytotoxic responses induced by an Ad5/HIV vaccine were similar to those of progressors. (A, B) Summary data of the total cytotoxic response measured by GrB activity (circles, A) or infected CD4^+^ T-cell elimination (ICE) (diamonds, B) with day 6 (D#6) CD8^+^ T-cells derived from LTNP (red symbols, n = 7), progressors (blue symbols, n = 10), HIV-negative individuals (black symbols, n = 10) and Ad5/HIV vaccinees (green symbols, n = 31). Data are representative of at least two experiments. Comparisons were made using the Wilcoxon two-sample test. Horizontal lines indicate median values. Only P values referring to comparisons between the responses of vaccinees and the other groups are shown. (C) Using D#6 CD8^+^ T-cells, GrB target cell activity correlates directly with ICE (n = 58). Correlation was determined by the Spearman rank method.

We have previously observed large differences in cytotoxic capacity between LTNP and viremic or ART-suppressed progressors, however, these measurements have not been applied to vaccinees. In their current form, these assays require large numbers of PBMC. For this reason, cells from participants in two trials (HVTN 071 and 502) in which vaccinees were leukapheresed or gave large blood volumes after either 2 or 3 doses, respectively, of the Merck Ad5/HIV trivalent vaccine were used. We have observed in prior work that although some differences in the cytotoxic capacity of unstimulated HIV-specific CD8^+^ T-cells were detectable between groups of chronically infected patients, the differences were largest in a recall response after a 6-day incubation with infected CD4^+^ T-cells [8], [19]. This likely represents the time necessary for upregulation of perforin under conditions of low levels of antigen such as would be expected in both LTNP and vaccinees. HIV-specific CD8^+^ T-cell cytotoxic responses measured by granzyme (Gr) B target cell activity and infected CD4^+^ T-cell elimination (ICE) were readily detectable in vaccine recipients (Table S1, Figure S1, Figure 1). The recall cytotoxic responses mediated by the cells of vaccinees (median GrB activity 15.6%, range 3-37.7%; median ICE 32.6%, range 5.8-61.5%) were significantly higher than those observed in HIV-negative controls (median GrB activity 1.7%, range 0-7.46%, p<0.001; median ICE 0.29%, range 0-3.3%, p<0.001; Figure 1A, B). In comparisons with chronically HIV-infected patients (Table S2), cytotoxic responses of vaccinees were significantly lower than, and completely non-overlapping with, those of LTNP (median GrB activity 50.7%, range 42.9-63%, p<0.001; median ICE 82.5%, range 75.2-86.6%, p<0.001; Figure 1A, B). Their responses were, however, comparable to those of progressors (median GrB activity 16.55%, range 3.03-39%, p>0.5; median ICE 37.35%, range 3.9-56%, p>0.5; Figure 1A, B). HIV-specific CD8^+^ T-cell cytotoxic responses measured by GrB or ICE were strongly, directly correlated with each other (R = 0.92, p<0.001), as observed previously in chronically infected patients (Figure 1C) [8], [19].

### Numbers of immunizations and time from last vaccination did not correlate with the magnitude of HIV-specific CD8^+^ T-cell cytotoxic responses induced by an Ad5/HIV vaccine

**Figure 2:**
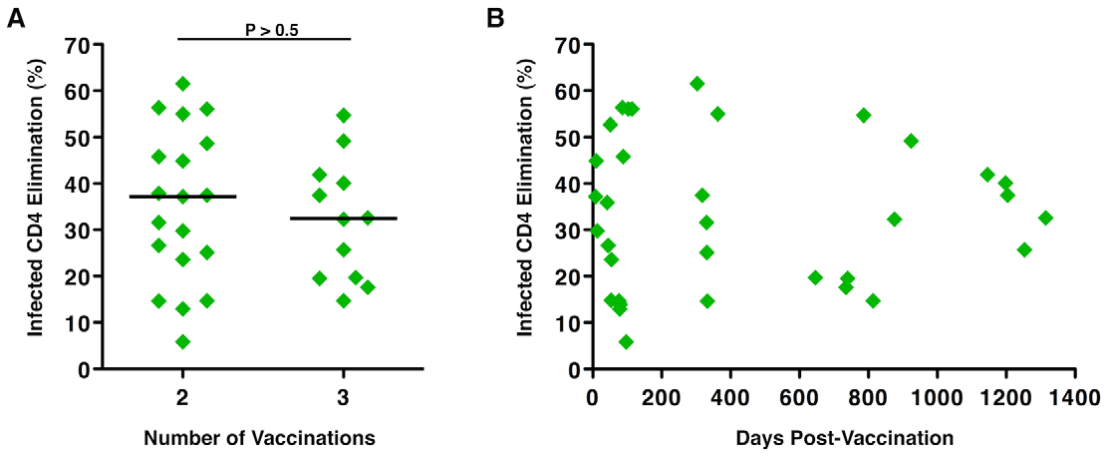
Numbers of immunizations and time from last vaccination did not correlate with the magnitude of Ad5/HIV vaccine-induced HIV-specific CD8^**+**^ T-cell cytotoxic responses. (A) ICE responses were compared by the Wilcoxon two-sample test between individuals who had received 2 (n = 19) versus 3 (n = 12) immunizations with the Ad5/HIV vaccine through protocols 071 or 502, respectively. Horizontal lines represent median values. (B) ICE responses did not correlate with the number of days following the last vaccination (p>0.5). Correlation was determined by the Spearman rank method.

The magnitude of vaccine-induced responses may be related to the potency and frequency of immunization, which may also influence durability of the response. In this study, the cytotoxic capacity of HIV-specific CD8^+^ T-cells derived from Ad5/HIV vaccine recipients was analyzed in the context of the number of immunizations and time since last immunization. No significant differences were observed in the magnitude of the CD8^+^ T-cell cytotoxic responses between individuals who had received 2 versus 3 immunizations (median ICE 37.2% versus 32.45%, respectively, p>0.5; Figure 2A). In addition, there was no correlation between the magnitude of ICE and the duration of time following the last immunization (p>0.5; Figure 2B). Some of the highest responses were observed 28 months after the last immunization. These results further support the relative immunogenicity of this vaccine construct, which induced significant cytotoxic responses following 2 doses, and are consistent with prior work showing little additional increase in the frequencies of HIV-specific CD8^+^ T-cells with a third vaccine dose [27]. These data also support the induction of memory HIV-specific CD8^+^ T-cells that persist and have retained expansion potential.

### Per-cell cytotoxic capacity of HIV-specific CD8^+^ T-cells induced by an Ad5/HIV vaccine was comparable to that of progressors

The differences in the magnitude of the cytotoxic responses observed in vaccine recipients compared to chronically infected patients might reflect very low precursor frequencies in the context of vaccination. This could result in comparatively lower responses after 6-day stimulation even if proliferative capacity remains intact. As an initial evaluation, we correlated the magnitude of the cytotoxic responses with the starting frequencies of CD8^+^ T-cells producing interferon-gamma (IFNγ) and/or IL2, which had been measured in intracellular cytokine staining (ICS) assays following 6-hour stimulation with HIV Nef, Gag or Pol peptide pools. PBMC were obtained from similar time points for use in both the ICS and cytotoxicity analyses. Since these responses had been measured against pools of potential T-cell epitopes (PTE) in the 071 study and pools of 15mer peptides overlapping in sequence by 11 amino acids in the 502 study, we evaluated them separately, even though these responses did not differ significantly between the vaccine groups by either method (median total IFNγ^+^ and/or IL2^+^ CD8^+^ T-cells 0.54% versus 0.57%, respectively, p>0.5; median IL2^+^ CD8^+^ T-cells 0.08% versus 0.07%, respectively, p>0.5; median total IFNγ^+^ and/or IL2^+^ CD4^+^ T-cells 0.12% versus 0.04%, respectively, p>0.5; median total IL2^+^ CD4^+^ T-cells 0.08% versus 0.0%, respectively, p = 0.26; data not shown). In the 071 study (n = 19), ICE did not correlate with either the total frequencies of IFNγ^+^ and/or IL2^+^ CD8^+^ T-cells (p = 0.2) or the IL2-secreting subset (p = 0.25; data not shown). It also did not correlate with the total frequencies of IFNγ^+^ and/or IL2^+^ CD4^+^ T-cells (p>0.5) or IL2^+^ CD4^+^ T-cells (p>0.5; data not shown). Except for a significant correlation between ICE and the frequencies of IL2^+^ CD4^+^ T-cells (r = 0.72, p = 0.04), similar results were observed in analyses performed among vaccine recipients in the 502 study (n = 10; data not shown). Therefore, among those responses evaluated in both assays, the frequencies of cytokine-producing HIV-specific CD8^+^ T-cells induced by this vaccine strategy were not predictive of killing capacity following 6-day stimulation with HIV-infected CD4^+^ T-cell targets.

**Figure 3:**
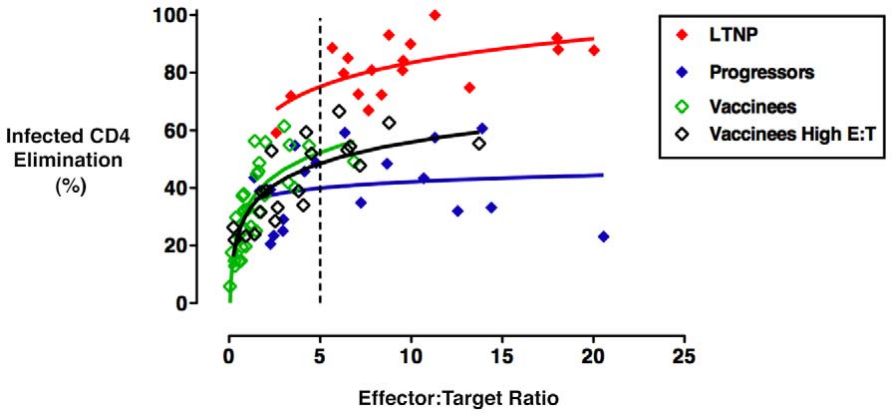
The HIV-specific CD8^**+**^ T-cells of Ad5/HIV vaccinees exhibited per-cell cytotoxic capacity that was significantly lower than that of LTNP but only somewhat higher than that of progressors. Cytotoxic responses mediated by day 6 CD8^+^ T-cells were measured by ICE for all individuals at a standard E:T ratio of 25∶1 or an increased E:T ratio of 50∶1 (total day 6 CD8^+^ T-cells to total CD4^+^ T-cell targets) and subsequently plotted against the true E:T ratios based on measurements of IFN-γ-secreting CD8^+^ T-cells and p24-expressing target cells, respectively. Curves represent trends for responses of LTNP (n = 18, red), viremic progressors (n = 19, blue), Ad5/HIV vaccinees measured at the standard E:T ratio of 25∶1 (n = 31, open green) and a subset of Ad5/HIV vaccinees also measured at an increased E:T ratio of 50∶1 (n = 20, open black). Regression analysis, analysis of covariance and repeated measures were used to quantify the differences in ICE among LTNP, progressors and Ad5/HIV vaccinees over the range of logged E:T ratios, at the median log E:T ratio of the combined groups and at the log E:T ratio of 5∶1. ICE of LTNP differed by constant amounts from that of vaccinees over the shared range of true E:T ratios measured with either standard (p<0.001) or increased (p<0.001) numbers of CD8^+^ T-cells. However, the differences between progressor and vaccinee ICE trend lines at standard (p = 0.02) and increased (p = 0.05) numbers of CD8^+^ T-cells depended on the value of the E:T ratio over the shared range. See text for more details.

To determine whether the low-level cytotoxic responses for the vaccine group relative to those of LTNP were due solely to diminished numbers of cells following the 6-day stimulation or also due to reduced per-cell killing capacity, we further analyzed the cytotoxic responses in the context of the true effector-to-target (E:T) ratios. For this analysis, ICE responses were measured at a standard E:T ratio of 25∶1 (total day 6 CD8^+^ T-cells to total CD4^+^ T-cell targets) for all individuals, and additionally at an increased E:T ratio of 50∶1 (by doubling the numbers of plated CD8^+^ T-cells) in 20 vaccinees from whom greater numbers of PBMC were available. These responses were then plotted against the measured HIV-specific E:T ratios, as previously described (see Methods) [8], [19]. Curves fit to the responses of the vaccinee group at standard and increased E:T ratios were overlaid on historical data generated with the cells of LTNP and viremic progressors and were analyzed by regressing ICE responses on the log of the true E:T ratios (Figure 3). Cytotoxic responses of LTNP were significantly greater than those of the vaccine recipients measured with both standard (n = 31) and increased (n = 20) CD8^+^ T-cell numbers by a constant 23% (p<0.001) and 27% (p<0.001), respectively, over the shared E:T ratios (Figure 3). In contrast, the differences between vaccinees and progressors at both standard (p = 0.02) and increased (p = 0.05) CD8^+^ T-cell numbers varied with the value of the E:T ratio and were not constant over their shared range. For example, the vaccinee curve summarizing responses measured with increased CD8^+^ T-cell numbers was similar to that of progressors at the median E:T ratio of 3.1∶1 (p = 0.17), but was significantly, yet modestly, greater at an E:T of 5∶1 (8.6%, p = 0.02; Figure 3). In summary, the per-cell cytotoxic capacity of HIV-specific CD8^+^ T-cells derived from vaccine recipients was consistently and significantly lower than that of LTNP across a broad range of shared E:T ratios, but was similar to, or only modestly greater than, the per-cell cytotoxic capacity of progressors.

### HIV-specific CD8^+^ T-cell proliferation and perforin expression correlate with cytotoxic capacity in recipients of an Ad5/HIV vaccine

The HIV-specific CD8^+^ T-cell cytotoxic responses of recipients of this immunogenic Ad5/HIV vaccine could be distinguished from the responses of HIV-negative individuals and LTNP, but were more comparable to those of progressors. We had previously shown in the setting of chronic infection that cytotoxicity directly correlated with proliferative capacity and expression of the cytotoxic proteins contained within cytotoxic granules [8], [19]. Given these findings, we explored the proliferative capacity and perforin expression of the HIV-specific CD8^+^ T-cells of vaccinees.

**Figure 4:**
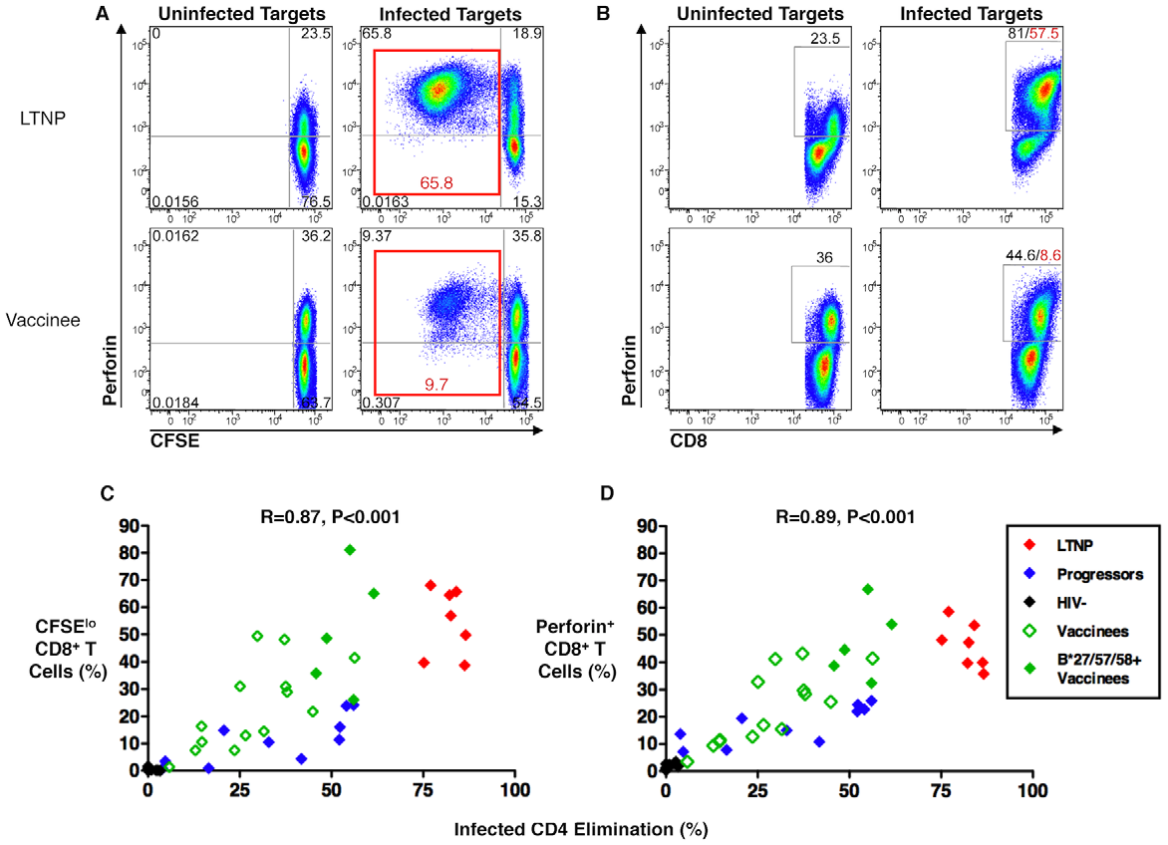
HIV-specific CD8^**+**^ T-cell proliferation and perforin expression correlated with cytotoxic capacity in recipients of an Ad5/HIV vaccine. (A, B) Flow cytometry plots of a representative LTNP (top row) and Ad5/HIV vaccine recipient (bottom row) depicting CD8^+^ T-cell proliferation (A) and CD8^+^ T-cell perforin expression (B) following 6-day stimulation of CFSE-labeled PBMC with uninfected (left columns) or HIV_SF162_-infected (right columns) autologous CD4^+^ T-cell targets. Plots are gated on CD8^+^ T-cells. Net HIV-specific CD8^+^ T-cell proliferation (sum of upper left and lower left quadrants in A, right column) and net CD8^+^ T-cell perforin expression (as determined by subtracting background perforin expression measured in cells that had been stimulated with uninfected targets; B, left column) are shown in red font. (C, D) Summary data of CD8^+^ T-cell proliferation (C) and perforin expression (D) are shown for LTNP (red symbols, n = 7), progressors (blue symbols, n = 10), HIV-negative individuals (black symbols, n = 10) and Ad5/HIV vaccinees (green symbols, n = 19), including 5 individuals carrying the protective HLA class I alleles B*27, B*57 or B*58 (solid green symbols). Data are representative of at least two experiments. Statistical analyses were performed using the Spearman correlation.

CD8^+^ T-cells were CFSE-labeled and incubated for 6 days in parallel with non-labeled cells that were to be used as effectors in the cytotoxicity assays. Following a 6-day incubation with autologous HIV-infected CD4^+^ T-cell targets, the CFSE-labeled cells were fixed, stained for perforin and analyzed by flow cytometry for proliferation and intracellular perforin expression (Figure 4A–D). Consistent with prior work, CD8^+^ T-cell proliferation and perforin expression were strongly directly correlated with each other (R = 0.96, p<0.001; data not shown). In analyses with cells from all patient groups, including the cells of the recipients of the Ad5/HIV vaccine, ICE was strongly directly correlated with both CD8^+^ T-cell proliferation (R = 0.87, p<0.001) and perforin expression (R = 0.89, p<0.001; Figure 4C, D). Focusing the analysis on the responses of vaccine recipients, CD8^+^ T-cell proliferation and perforin expression were again strongly directly correlated with each other (R = 0.96, p<0.001; data not shown) and with ICE (R = 0.74, p = 0.001 and R = 0.81, p<0.001, respectively; Figure 4C, D). These findings suggest that the same cascade of events that has been associated with maximal cytotoxic capacity in chronically infected patients also occurs in vaccine recipients. Interestingly, however, the proliferative responses and perforin expression of the CD8^+^ T-cells of some of the vaccine recipients were disproportionately high for the degree of cytotoxicity and overlapped the responses of LTNP (Figure 4C, D). That is, their cytotoxic responses were lower than might be predicted by HIV-specific CD8^+^ T-cell proliferative capacity and intracellular perforin expression, which contrasts with observations made in chronically infected patients. These data suggest that although proliferative capacity and perforin expression are correlated with cytotoxic capacity, these correlations are not as tight as those observed in chronic infection. Importantly, the proliferative capacity and perforin expression of CD8^+^ T-cells of some vaccinees overlapped with those of LTNP although there were considerable differences in cytotoxic capacity.

### HIV-specific CD8^+^ T-cell cytotoxic capacity in Ad5/HIV vaccine recipients was higher among individuals with HLA class I alleles associated with nonprogressive HIV infection

**Figure 5:**
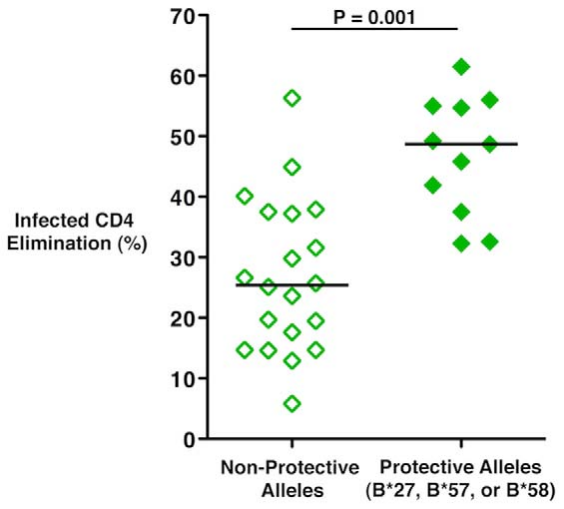
HIV-specific CD8^**+**^ T-cell cytotoxic capacity in Ad5/HIV vaccine recipients was higher among individuals with protective HLA class I alleles. ICE responses mediated by day 6 CD8^+^ T-cells of vaccine recipients who possess the HLA class I alleles B*27, B*57 or B*58 that have been shown to be associated with nonprogressive HIV infection (n = 11) versus those of vaccine recipients not possessing these protective alleles (n = 20) was compared by the Wilcoxon two-sample test. Horizontal lines represent median values. Data are representative of at least two experiments.

Given the preliminary data showing a trend towards an association between HLA haplotype and viral load in vaccinee cases of the Step trial (Nicole Frahm, personal communication), it was of interest to examine the cytotoxic capacity of vaccinees in the present study when stratified by HLA type. Interestingly, the cytotoxic responses of vaccine recipients carrying HLA class I alleles that have been associated with nonprogressive HIV infection, e.g., B*27, B*57 and B*58 (n = 11), were significantly greater than those of individuals not possessing these alleles (n = 20; median ICE 48.7% versus 25.4%, respectively, p = 0.001; Figure 5). Importantly, none of the individuals bearing these protective alleles exhibited a low response. These results suggest that, in contrast to patients not expressing protective alleles, vaccination in individuals carrying these alleles primes for greater HIV-specific CD8^+^ T-cell cytotoxic capacity.

## Discussion

In the present study, we examined the cytotoxic capacity of HIV-specific CD8^+^ T-cells, in response to primary autologous HIV-infected CD4^+^ T-cell targets, in samples from HIV-negative individuals vaccinated with a replication-incompetent adenovirus serotype 5 HIV vaccine. In prior studies, immunization with this vaccine did not diminish the rate of HIV infection compared to placebo recipients, nor did it lead to reductions in plasma HIV RNA levels among newly infected persons [26]. In our analyses, readily detectable HIV-specific CD8^+^ T-cell cytotoxic responses were observed in vaccinees based on measurements of GrB target cell activity and infected CD4^+^ T-cell elimination. Significant responses were present a median of 331 days following the last immunization, confirming that long-lived memory cells had been induced with this vaccine strategy. However, the recall cytotoxic capacity of the HIV-specific CD8^+^ T-cells of vaccinees was modest and overlapped with that of progressors. The magnitude of the cytotoxic responses was not related to the number of vaccinations, nor did it correlate with the percentages of cytokine-secreting T-cells determined by ICS assays. Importantly, we did observe higher cytotoxic responses in vaccine recipients carrying HLA class I alleles that have been associated with immune control over HIV replication, HLA B*27, B*57 or B*58.

Given the lack of an association between traditional measures of HIV-specific T-cell frequencies such as ELISPOT or ICS and immunologic control of HIV after vaccination, there has been increasing interest in measurements of other HIV-specific T-cell functions. Significant activity was observed with viral inhibition assays in recipients of a DNA prime/recombinant Ad5 boost vaccine regimen [32], [33]. Spentzou et al. observed relatively low but detectable inhibition by CD8^+^ T-cells from 7 participants that had received a DNA-recombinant Ad5 prime-boost regimen [32]. Freel et al. observed low virus inhibition in 40 participants vaccinated with a similar DNA prime/recombinant Ad5 boost regimen [33]. Many of these responses were below the level of detection and the responses of vaccinees were below those of progressors and nonprogressors. Of note, the response of chronic progressors was similar to those of virus controllers [33]. Differences between these results and ours may be attributed to differences in vaccine regimen and cohort selection criteria [8], [19], [33]. In addition, they may be attributable to the assays used. Although granule exocytosis-mediated killing may be an important contributor in virus inhibition assays, the latter may also measure the effects of CD8^+^ T-cell proliferation, chemokine or suppressor factor secretion, or cytotoxicity mediated by other mechanisms. Nonetheless, it will be important over the coming years to follow each of these assays for their ability to predict vaccine-induced immunologic control of HIV and for potential clues regarding the mechanism.

Although some HIV-specific CD8^+^ T-cell cytotoxic capacity was induced by the vaccine approach in the current study, the magnitude necessary for immunologic restriction of HIV remains unclear. Since participants were not infected after vaccination, it was reasonable to suspect their precursor frequencies were most likely lower than those of chronically infected patients. Although this vaccine approach induced pre-challenge levels of Mamu A01-p11CM MHC tetramer^+^ CD8^+^ T-cells in the peripheral blood of rhesus macaques that reached 2% in the Ad5/SIV only arm and levels ranging from 5–25% when preceded by a DNA/CRL1005 prime, the Ad5/HIV vaccine induced only a median response of 0.4–1% HIV-specific CD8^+^ T-cells based upon ICS in humans in a prior study and 0.5–0.6% in the present study [25], [27]. However, it is clear that the modest cytotoxic capacity observed in vaccinees was not simply due to low frequencies of HIV-specific CD8^+^ T-cells. The frequencies of HIV-specific CD8^+^ T-cells did not correlate with cytotoxic capacity. Furthermore, some of the individuals with the lowest frequencies of HIV-specific T-cells exhibited the highest CD8^+^ T-cell cytotoxic responses several years after vaccination.

Another potential factor contributing to the diminished HIV-specific CD8^+^ T-cell-mediated cytotoxicity observed in vaccinees relative to LTNP might relate to response breadth. That is, there may be additional CD8^+^ T-cell responses in LTNP targeting proteins outside of the 3 genes included in the vaccine, which might have led to more efficient elimination of HIV-infected CD4^+^ T-cell targets. This seems unlikely to be the case, however, since the bulk of the cytokine secretory and proliferative responses measured in LTNP have been primarily directed against highly conserved epitopes contained within Nef, Gag and Pol, with only minimal contribution made by responses targeting other gene products [2], [15], [21], [34].

In order to investigate the cytotoxic responses of the various groups in greater detail, we analyzed them in the context of the true measured E:T ratios following 6-day re-stimulation and found that the per-cell killing capacity for the group of vaccine recipients again remained only slightly higher than that of progressors, but significantly lower than that of LTNP. Although it is not necessarily expected that the CD8^+^ T-cells of vaccinees would achieve the cytotoxic capacity of chronically infected LTNP, the results of the present study suggest that the human immune response is capable of higher responses. The observation that vaccinee per-cell cytotoxic capacity increased at higher E:T ratios and became more divergent from that of progressors also suggests that induction of higher cytotoxic responses might be attainable with improved vaccine design strategies. These results suggest a threshold level of cytotoxic capacity might have been achieved only in a small subset of patients with protective alleles in this vaccination scheme. Further investigation of the cytotoxic responses directed against other viral infections that have been cleared or are controlled by the host may provide a better context in which to interpret the responses measured in this study. Most importantly, further evaluation of vaccinee cases may reveal whether these measurements of HIV-specific CD8^+^ T-cell cytotoxic capacity can accurately predict immune control over HIV following vaccination.

In the present study, we did observe a clear effect of protective HLA alleles in priming HIV-specific CD8^+^ T-cell cytotoxic capacity. However, whether these alleles, in the context of vaccination, are associated with qualitatively different CD8^+^ T-cell responses or immunologic control has been examined in some prior work with mixed results [28], [33]. Preliminary data from the Step study suggested that, after HIV infection, vaccinees with the protective HLA alleles B*57, B*58 and B*27 may have lower HIV RNA levels compared to those with protective alleles that received placebo (Nicole Frahm, personal communication). However, in a subsequent analysis, although those with protective alleles had lower viral loads overall, the difference between placebo and vaccinee cases with protective alleles did not achieve statistical significance (Nicole Frahm, personal communication). Kaslow et al observed that CD8^+^ T-cells restricted by protective alleles dominate the response to vaccination with an ALVAC-HIV recombinant canarypox [28]. In addition, Freel et al. observed some increased HIV inhibition in cells from vaccinees with B*27 or B*57 compared to those who lacked these alleles. However, this was only true of NL4-3 and WEAU viruses [33]. Taken together, these data suggest that protective alleles may function to more efficiently prime HIV-specific CD8^+^ T-cell cytotoxic capacity. The precise mechanism underlying this association, however, is currently unclear. CD8^+^ T-cell responses restricted by HLA B27 and 57 predominate during acute infection [35] and are more likely to maintain the capacity to proliferate after prolonged infection than responses restricted by other alleles [34], [36]. Enhanced induction of CD8^+^ T-cell proliferative responses leading downstream to increased cytotoxic capacity might result from a greater ability of infected cells to stimulate naïve CD8^+^ T-cells. Alternatively, it might result from an enhanced ability of CD8^+^ T-cells to respond to infected cells due to interactions related to killer immunoglobulin-like receptor (KIR) ligation, co-stimulatory signal requirements, or a diverse naïve T-cell repertoire. In any event, whether the increased cytotoxic capacity observed in the present study translates into better immunologic control will need to be monitored in future trials.

Although the HIV-specific CD8^+^ T-cell cytotoxic capacity of vaccinees was less than that observed among LTNP, the cells of some vaccinees rapidly expanded and produced perforin in response to HIV-infected T-cells. In chronic infection, we have observed a very tight correlation between proliferative capacity and perforin expression as we did in the present study [21]. However, a range of proliferative responses and perforin expression was observed in vaccinees that, in some cases, overlapped those of LTNP even though these cells did not exhibit cytotoxic responses of similar magnitudes as those of LTNP. It is unlikely that such a large fraction of vaccinees would exhibit immunologic control upon infection. Thus, proliferation or perforin expression, at least under these experimental conditions, are unlikely to be better predictors of immunologic control than cytotoxic capacity in the context of vaccination. The observation of cells with proliferative potential and the ability to make perforin similar to those of LTNP but with only modest cytotoxic capacity may relate to vaccine vector, dose, vaccine administration schedule and timing of post-vaccination PBMC collection for use in the immunologic assays. These might have an impact on the pattern of differentiation or maturation of HIV-specific CD8^+^ T-cells, which could influence direct measures of killing capacity. Additional studies are currently under way to further characterize the functional capabilities of these cells.

Although many challenges lie ahead, it appears possible that T-cell based immunogens may provide some activity to reduce viral load upon infection. Currently, antibody-based vaccine approaches are receiving increased emphasis with the reported reduction in HIV acquisition observed in the RV144 trial [37]. However, an effective T-cell response would potentially complement an effective humoral immune response by reducing viral load in vaccinees that become infected [38]. It may also have the effect of reducing the transmission of antibody resistant mutants. Immunologic control in chronically infected LTNP is durable and has lasted more than 25 years in many cases (reviewed in [1]). In addition, many patients lack known protective MHC alleles suggesting these are not an absolute requirement for durable immunologic control [1]. Evidence of durable immunologic control that extends beyond known protective alleles has also been provided by recent studies in the SIV infection model. Cellular immune responses elicited by infection with recombinant rhesus cytomegalovirus encoding SIV antigens can provide resistance to SIV infection on repeated limiting-dose intrarectal challenge [39]. Ongoing studies are examining whether cytotoxic capacity is an important mechanism of immune control in the rhesus macaque-SIV infection model (Daniel Mendoza, unpublished observations). Importantly, our assay will be applied to participants in the Step study who subsequently acquired HIV infection post-vaccination, including a group of individuals who are restricting HIV replication to the limits of detection in currently available viral load assays. These types of analyses will begin to determine the utility of measurements of cytotoxic capacity in predicting vaccine efficacy, and provide further insight into the mechanisms of immunologic control of HIV.

## Supporting Information

Figure S1 HIV-specific CD8^+^ T-cell cytotoxic responses were measured by granzyme B target cell activity and infected CD4^+^ T-cell elimination in chronically infected patients and Ad5/HIV vaccine recipients. (A) Following incubation with day 6 autologous CD8^+^ T-cells, granzyme (Gr) B activity in uninfected (left column) or HIV-infected CD4^+^ T-cell lymphoblast targets (right column) is shown in a representative LTNP (top row) and an Ad5/HIV vaccine recipient (bottom row). Plots are gated on live targets based on staining with a LIVE/DEAD Fixable Violet Stain (see Methods). Net GrB target cell activity after subtracting background values (i.e., responses in uninfected targets, left column) is shown in red font. (B) Cells from A after fixation, permeabilization and staining for CD4 and intracellular p24 expression. Quadrants indicate percentages of gated targets. Infected CD4 elimination (ICE) is shown in red font, which was calculated with p24^+^ targets (sum of upper quadrants) as described in the Methods.
(TIFF)

Table S1 Ad5/HIV vaccine recipient characteristics.
(DOCX)

Table S2 Characteristics of HIV-infected patients.
(DOCX)
