# Supplementary figures and images for: Trivalent Adenovirus Type 5 HIV Recombinant Vaccine Primes for Modest Cytotoxic Capacity That Is Greatest in Humans with Protective HLA Class I Alleles
Source: PLoS Pathog. 2011 Feb 24;7(2):e1002002. doi: 10.1371/journal.ppat.1002002 (PMC3044701; doi:10.1371/journal.ppat.1002002)

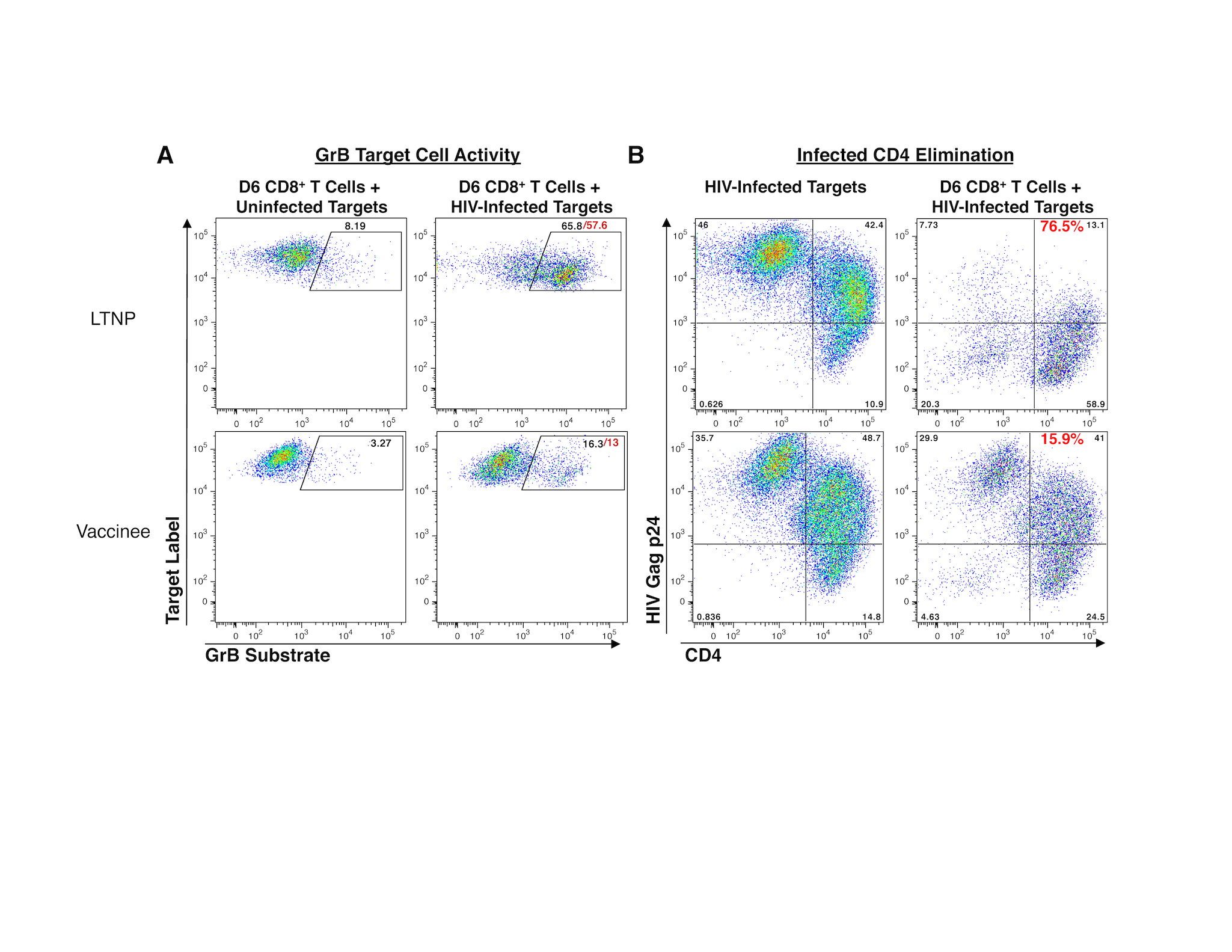

Supplement: Figure S1 — HIV-specific CD8+ T-cell cytotoxic responses were measured by granzyme B target cell activity and infected CD4+ T-cell elimination in chronically infected patients and Ad5/HIV vaccine recipients. (A) Following incubation with day 6 autologous CD8+ T-cells, granzyme (Gr) B activity in uninfected (left column) or HIV-infected CD4+ T-cell lymphoblast targets (right column) is shown in a representative LTNP (top row) and an Ad5/HIV vaccine recipient (bottom row). Plots are gated on live targets based on staining with a LIVE/DEAD Fixable Violet Stain (see Methods). Net GrB target cell activity after subtracting background values (i.e., responses in uninfected targets, left column) is shown in red font. (B) Cells from A after fixation, permeabilization and staining for CD4 and intracellular p24 expression. Quadrants indicate percentages of gated targets. Infected CD4 elimination (ICE) is shown in red font, which was calculated with p24+ targets (sum of upper quadrants) as described in the Methods. (TIFF) [file ppat.1002002.s001.tiff]
