# Supplementary material for: Trivalent Adenovirus Type 5 HIV Recombinant Vaccine Primes for Modest Cytotoxic Capacity That Is Greatest in Humans with Protective HLA Class I Alleles
Source: PLoS Pathog. 2011 Feb 24;7(2):e1002002. doi: 10.1371/journal.ppat.1002002 (PMC3044701; doi:10.1371/journal.ppat.1002002)
Supplement: Table S1 — Ad5/HIV vaccine recipient characteristics. (DOCX) [file ppat.1002002.s002.docx]

**Supplemental Table 1. Ad5/HIV Vaccine Recipient Characteristics**

| **Vaccinee**  **Number** | **Number of**  **Vaccinations** | **Leukapheresis**  **Days Post Vx** | **HLA Class I Alleles**  **A, B, C** | | |
| --- | --- | --- | --- | --- | --- |
| **HVTN 071** | | | | | |
| 125360010 | 2 | 103 | 2 | 15,27 | 1,3 |
| 126360051 | 2 | 318 | 2,31 | 7,40 | 3,7 |
| 121360037 | 2 | 78 | 24,30 | 42,53 | 4,17 |
| 125360049 | 2 | 333 | 30,33 | 8,14 | 7,8 |
| 125360102 | 2 | 76 | 2,24 | 14,35 | 4,8 |
| 123360032 | 2 | 97 | 11,68 | 15,55 | 3 |
| 123360040 | 2 | 88 | 1,3 | 40,57 | 3,6 |
| 123360058 | 2 | 86 | 1,2 | 8,40 | 3,7 |
| 121360020 | 2 | 356 | 23,74 | 18,58 | 7 |
| 126360047 | 2 | 303 | 2,26 | 27,38 | 1,12 |
| 126360104 | 2 | 330 | 24,68 | 44,51 | 7,14 |
| 126360012 | 2 | 45 | 25,32 | 18,40 | 3,12 |
| 126360064 | 2 | 54 | 11,33 | 14,52 | 8,12 |
| 126360070 | 2 | 331 | 24,30 | 13,40 | 3,6 |
| 126360129 | 2 | 10 | 2,3 | 52,53 | 4,16 |
| 126360133 | 2 | 13 | 1,24 | 7,49 | 7 |
| 126360157 | 2 | 8 | 1,68 | 8,52 | 7,12 |
| 121360014 | 2 | 357 | 2,68 | 44,58 | 4,7 |
| 126360176 | 2 | 21 | 3,68 | 13,18 | 6,12 |
| **HVTN 502** | | | | | |
| 140700145 | 3 | 828 | 2,68 | 40,51 | 3,15 |
| 109700037 | 3 | 661 | 1,2 | 8,44 | 5,7 |
| 164700148 | 3 | 864 | 2 | 35,57 | 4,7 |
| 109700099 | 3 | 1204 | 26,66 | 52,57 | 6,12 |
| 109700130 | 3 | 799 | 2,11 | 51,57 | 6,15 |
| 109700045 | 3 | 737 | 3,11 | 7,55 | 3,7 |
| 107700010 | 3 | 742 | 1 | 8,35 | 4,7 |
| 171700069 | 3 | 1253 | 3,30 | 7,13 | 6,7 |
| 115700048 | 3 | 930 | 1,2 | 27,57 | 1,7 |
| 107700054 | 3 | 1315 | 2,11 | 27,35 | 2,4 |
| 107700062 | 3 | 1198 | 2,24 | 49,51 | 7,14 |
| 107700049 | 3 | 1146 | 2,32 | 27,52 | 2,12 |
