# Supplementary material for: Trivalent Adenovirus Type 5 HIV Recombinant Vaccine Primes for Modest Cytotoxic Capacity That Is Greatest in Humans with Protective HLA Class I Alleles
Source: PLoS Pathog. 2011 Feb 24;7(2):e1002002. doi: 10.1371/journal.ppat.1002002 (PMC3044701; doi:10.1371/journal.ppat.1002002)
Supplement: Table S2 — Characteristics of HIV-infected patients. (DOCX) [file ppat.1002002.s003.docx]

**Supplemental Table 2. Characteristics of HIV-Infected Patients**

| **Patient Number** | **Diagnosis Year** | **CD4 T Cells**  **(Cells/mL)** | **CD8 T Cells**  **(Cells/mL)** | **HIV-1 RNA**  **(Copies/mL)** | **HLA Class I**  **A, B, C** | | |
| --- | --- | --- | --- | --- | --- | --- | --- |
| **LTNP** | | | | | | | |
| 4 | 1985 | 1063 | 1088 | <50 | 1,31 | 8,57 | 6,7 |
| 8 | 1985 | 664 | 1120 | <50-930 | 11,23 | 44,57 | 4,6 |
| 33 | 1995 | 955 | 881 | <50 | 2,30 | 13,57 | 6 |
| 38 | 1990 | 1329 | 1243 | <50 | 2,24 | 44,57 | 5,6 |
| 65 | 1993 | 865 | 388 | <50 | 30,74 | 14,57 | 2,8 |
| 68 | 1986 | 1362 | 1055 | <50 | 3,29 | 57,81 | 18 |
| 73 | 1991 | 801 | 1012 | <50 | 2,3 | 7,57 | 6,7 |
| **Viremic Progressors** | | | | | | | |
| 107 | 1987 | 445 | 1674 | 120291 | 3 | 40,57 | 3,7 |
| 131 | 1989 | 238 | 1017 | 85981 | 2,11 | 35,57 | 4,6 |
| 139 | 1993 | 453 | 861 | 78984 | 2,32 | 27,35 | 1,4 |
| 148 | 1999 | 243 | 757 | 94919 | 2,3 | 27,42 | 2,17 |
| 149 | 1991 | 739 | 979 | 30733 | 3,24 | 7 | 7,15 |
| 103 | 1991 | 457 | 977 | 5054 | 2,11 | 55,57 | 3,6 |
| **Treated Progressors (Viral Load <50 Copies/mL)** | | | | | | | |
| 127 | 1994 | 720 | 702 | <50 | 3,24 | 7,18 | 7 |
| 141 | 2001 | 408 | 553 | <50 | 2,24 | 35,49 | 3,7 |
| 174 | 1992 | 675 | 459 | <50 | 33,74 | 42,53 | 4,17 |
| 194 | 1987 | 709 | 902 | <50 | 2,30 | 40,51 | 3,16 |
